# One-step direct conversion of methane to methanol with water in non-thermal plasma

**DOI:** 10.1038/s42004-022-00735-y

**Published:** 2022-10-10

**Authors:** Wenfei Bi, Yu Tang, Xuemei Li, Chengyi Dai, Chunshan Song, Xinwen Guo, Xiaoxun Ma

**Affiliations:** 1 School of Chemical Engineering, International Science & Technology Cooperation Base for Clean Utilization of Hydrocarbon Resources, Chemical Engineering Research Center of the Ministry of Education for Advanced Use Technology of Shanbei Energy, Collaborative Innovation Center for Development of Energy and Chemical Industry in Northern Shaanxi, Northwest University, Xi’an, 710069 China; 2 Department of Chemistry, Faculty of Science, The Chinese University of Hong Kong, Shatin, NT, Hong Kong, China; 3 State Key Laboratory of Fine Chemicals, PSU-DUT Joint Center for Energy Research, School of Chemical Engineering, Dalian University of Technology, Dalian, 116024 China

**Keywords:** Energy, Natural gas, Heterogeneous catalysis

## Abstract

Achieving methane-to-methanol is challenging under mild conditions. In this study, methanol is synthesized by one-step direction conversion of CH_4_ with H_2_O at room temperature under atmospheric pressure in non-thermal plasma (NTP). This route is characterized by the use of methane and liquid water as the reactants, which enables the transfer of the methanol product to the liquid phase in time to inhibit its further decomposition and conversion. Therefore, the obtained product is free of carbon dioxide. The reaction products include gas and liquid-phase hydrocarbons, CO, CH_3_OH, and C_2_H_5_OH. The combination of plasma and semiconductor materials increases the production rate of methanol. In addition, the addition of Ar or He considerably increases the production rate and selectivity of methanol. The highest production rate of methanol and selectivity in liquid phase can reach 56.7 mmol g_cat_^−1^ h^−1^ and 93%, respectively. Compared with the absence of a catalyst and added gas, a more than 5-fold increase in the methanol production rate is achieved.

## Abstract (web summary)

Direct conversion of methane to methanol has great industrial relevance, but required high reaction temperatures can lead to overoxidation of methane to carbon dioxide. Here, methanol is synthesized by one-step reaction of methane and water over a TiO_2_ catalyst in a non-thermal plasma at room temperature and atmospheric pressure, which allows to isolate carbon dioxide-free methanol.

## Introduction

Methanol is an important raw material for chemical production, and the direct conversion of methane into methanol has commercial value. Currently, the industrial process to synthesize methanol involves the use of syngas^1^, which is usually prepared by methane reforming^2,3^, but the cost of this process is high^4^. The direct one-step oxidation of methane to methanol is more advantageous than the syngas route. Noble metals (e.g., Au, Pd, and Rh) have been reported to exhibit excellent catalytic properties for the conversion of methane^5–10^. Agarwal et al.^5^ oxidized methane to methanol using Pd–Au colloidal nanoparticles in the presence of H_2_O_2_ and O_2_. Xiao et al.^7^ designed a “molecular fence” catalyst synthesized by fixation of AuPd alloy nanoparticles within aluminosilicate zeolite crystals followed by modification of the external zeolite surface with organosilanes. Methane was converted into methanol with high efficiency, with a production rate of 91.6 mmol g_AuPd_^−1^ h^−1^. In addition, some non-noble metals have also been reported to show potential for this reaction. Inspired by the biocatalysis of methane monooxygenase (MMOS)^11,12^, Grundner et al.^13^ prepared a Cu-MOR catalyst by ion exchange, with active sites similar to those present in MMOS. The conversion of methane to methanol was realized by multiple reaction steps (activating active sites under an O_2_ atmosphere, methane reaction, and methanol hydrolysis). Except for intermittent reactions, Narsimhan et al.^14^ reported the simultaneous passage of methane, oxygen, and water into the reactor for the reaction. Although the reported conversion was not high, the oxidation of methane to methanol under continuous conditions was realized for the first time.

Besides the complexity of the operation process, a high reaction temperature is employed due to the intrinsic characteristics of methane, which is difficult to activate^15–17^, and the oxidation of the product methanol is easier than that of methane, typically leading to the continuous oxidation of methanol to CO_2_ under the reaction conditions. Hence, unsatisfactory results are obtained^18,19^. NTP is a form of plasma with the characteristics of low temperature and high-energy electrons^20^. Therefore, the introduction of NTP into the reaction system can convert some difficult-to-activate molecules into active groups^21^ and considerably accelerate the reaction speed. Nevertheless, the inhibition of the over-oxidation of methane is still an urgent issue.

**Fig. 1:**
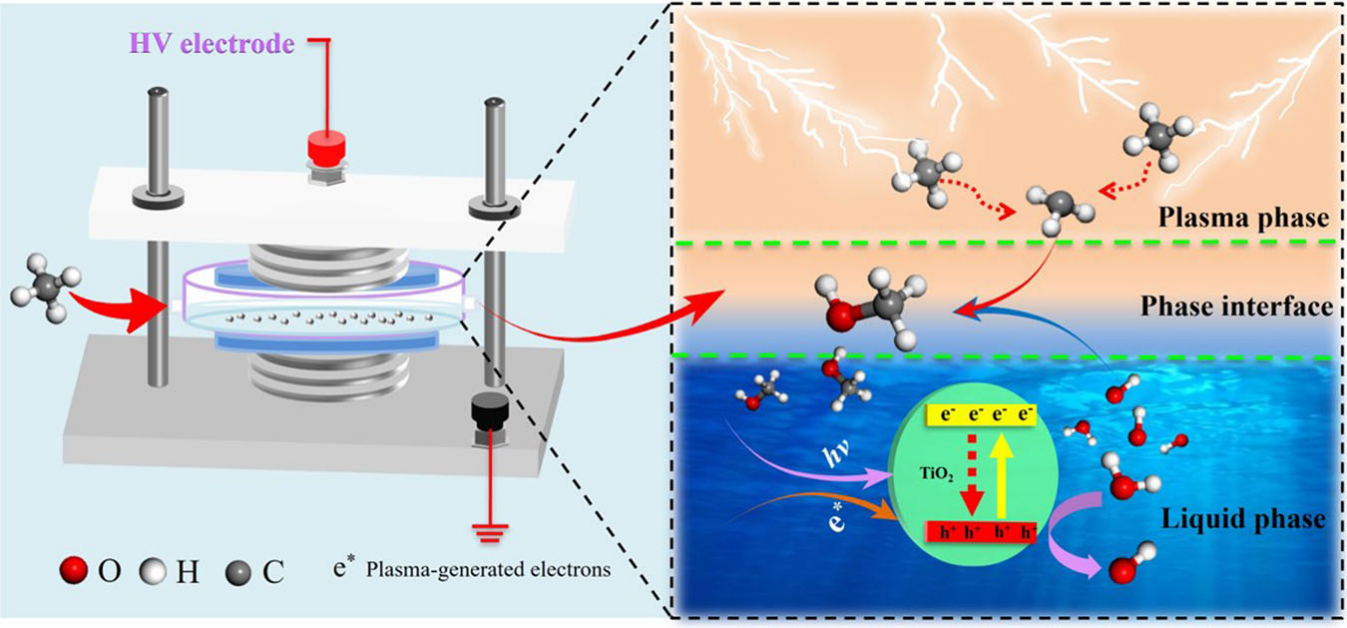
Schematic of the plasma system. The reaction between CH_4_ and H_2_O to afford methanol is performed in a dielectric barrier discharge (DBD) reactor.

In this study, a new route to synthesize methanol from methane using NTP is designed, which reasonably and completely exploits the catalytic characteristics of plasma. At room temperature under atmospheric pressure, the formation of methanol is realized by directly mixing CH_4_ and a weak oxidant H_2_O without cumbersome operational steps. The reaction between CH_4_ and H_2_O to afford methanol is performed in a dielectric barrier discharge (DBD) reactor (Fig. 1). The reaction environment involves three parts: the plasma phase, the liquid phase, and the plasma-liquid interface. Each of these parts corresponds to the formation of methyl radicals (CH_3_·), formation of hydroxyl radicals (·OH), and production of methanol. Methane in the plasma phase is activated by inelastic collision with high-energy electrons, becomes an excited state (CH_4_*), and is decomposed to methyl radicals (CH_3_·). These activated molecules and radicals contribute predominantly to methanol production. Liquid-phase H_2_O provides the required ·OH for the reaction. By constructing a TiO_2_ surface heterojunction in the liquid phase and using the photons and electrons^22^ produced in the plasma, the formation of hydroxyl radicals is significantly promoted. In addition, the liquid-phase environment is conducive to the instantaneous transfer of methanol produced at the liquid-plasma interface, inhibiting the decomposition and transformation of methanol.

## Results and discussion

### Catalytic performance

**Fig. 2:**
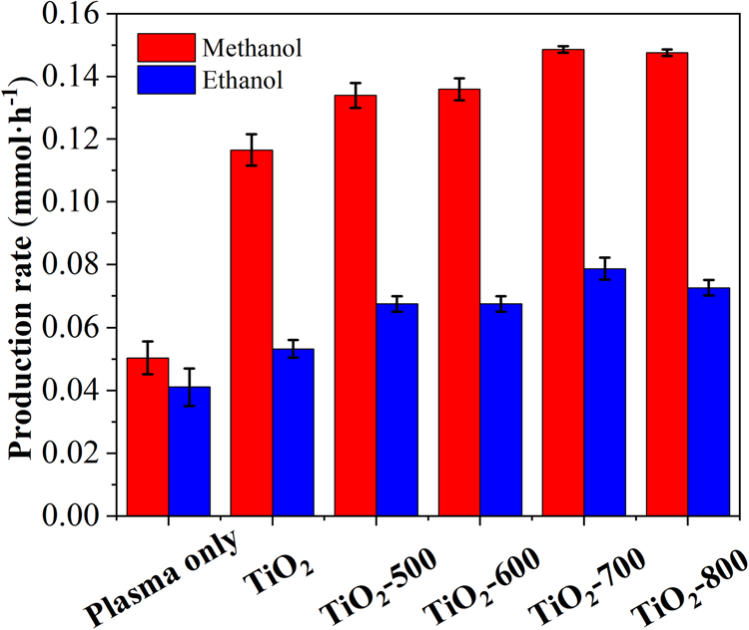
Alcohol production rate. Effects of different catalysts on the reaction (CH_4_ flow rate of 5 mL min^−1^, discharge power of 30 W, ca. 5 mg catalyst, error bars obtained from repeated three sets of experiments on the same catalyst).

Methanol can be produced in all experiments by this route, and it is crucial that CO_2_ is not produced. The presence of a small amount of methanol is detected under the plasma-only mode (Fig. 2). On the one hand, the CH_3_· produced by CH_4_ activation can directly react with H_2_O to form methanol; on the other hand, the plasma decomposes H_2_O molecules when they reach the phase interface, and the gas phase comprises a small amount of water vapor, which is also converted into ·OH by high-energy electrons. Clearly, the addition of the TiO_2_ catalyst in the DBD plasma enhances the production rate of methanol to 29.7 mmol g_cat_^−1^ h^−1^ (Supplementary Fig. 1). Compared with the absence of a catalyst, a 3-fold increase in the methanol production rate is achieved. The selectivity of gas phase and liquid phase products are shown in Table S1, it can be seen that 4.5–12.7% of methane is converted to methanol.

**Fig. 3:**
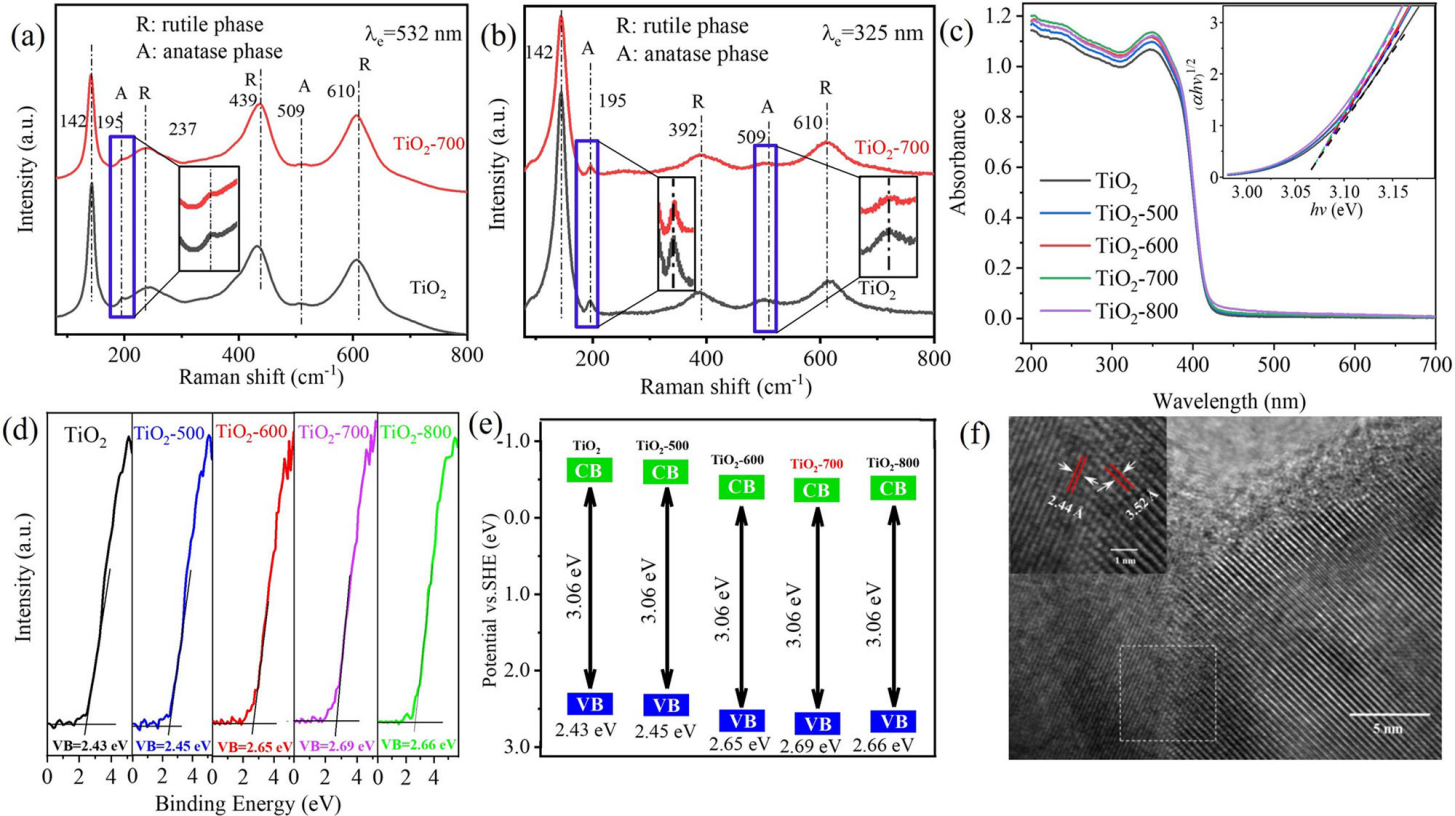
Spectra and HRTEM image of the samples. **a** Visible Raman spectra, **b** UV Raman spectra, **c** UV–Vis spectra, **d** XPS spectra showing the valence band (VB) levels, **e** schematic of the band-gap structure, and **f** HRTEM image of the TiO_2_-700 sample.

X-ray diffraction (XRD) was employed to characterize the chemical structures of a series of TiO_2_-t samples. Peaks at 25.3°, 37.8°, 48.0°, and 62.7° correspond to the characteristic diffraction peaks of anatase, while those at 27.4°, 36.1°, 41.2°, and 54.3° correspond to those of rutile. The XRD spectra reveal that the catalyst contains both anatase and rutile and that the rutile is the main crystal form (Supplementary Fig. 2). High-resolution transmission electron microscopy (HRTEM) reveals that the TiO_2_-700 shows crystal plane spacings at ~2.44 and 3.52 Å, corresponding to the (101) crystal plane of the rutile phase and the (101) crystal plane of the anatase phase (Fig. 3f), respectively. This is consistent with the XRD analysis. This catalyst is advantageous as the hybrid catalyst consisting of anatase and rutile can form a heterojunction. Hence, electrons and holes can be effectively separated and lead to better catalyst performance. The surface phase composition of TiO_2_ is the key factor that affects the catalytic performance^21^. Visible Raman and UV Raman spectra (Fig. 3a, b) reveal the presence of anatase and rutile phase junctions on the catalyst surface, and the characteristic peak value of anatase in the TiO_2_-700 sample decreases, indicating that the surface phase changes. The samples show similar band gaps in the UV–Vis spectra (Fig. 3c), but the light absorption intensity of TiO_2_ gradually increases with increasing treatment temperature, corresponding to the maximum at 700 °C. The X-ray photoelectron spectroscopy valence band (XPS-VB) spectra (Fig. 3d) reveal that the valence band energy of each sample after treatment gradually increases and that the peak value is attained at 700 °C. Figure 3e shows a schematic of the band-gap structure. The VB energy value of 2.43 eV for unprocessed raw powder TiO_2_ changes to 2.69 eV for TiO_2_ treated at 700 °C, indicative of the improved oxidation performance of the holes formed by the treated catalyst. The catalytic performance from experiments is consistent with the characterization results. With the increase in the treatment temperature, the production rate of methanol increases to the peak value and then decreases, and TiO_2_-700 exhibits excellent performance.

### Possible reaction pathways

**Fig. 4:**
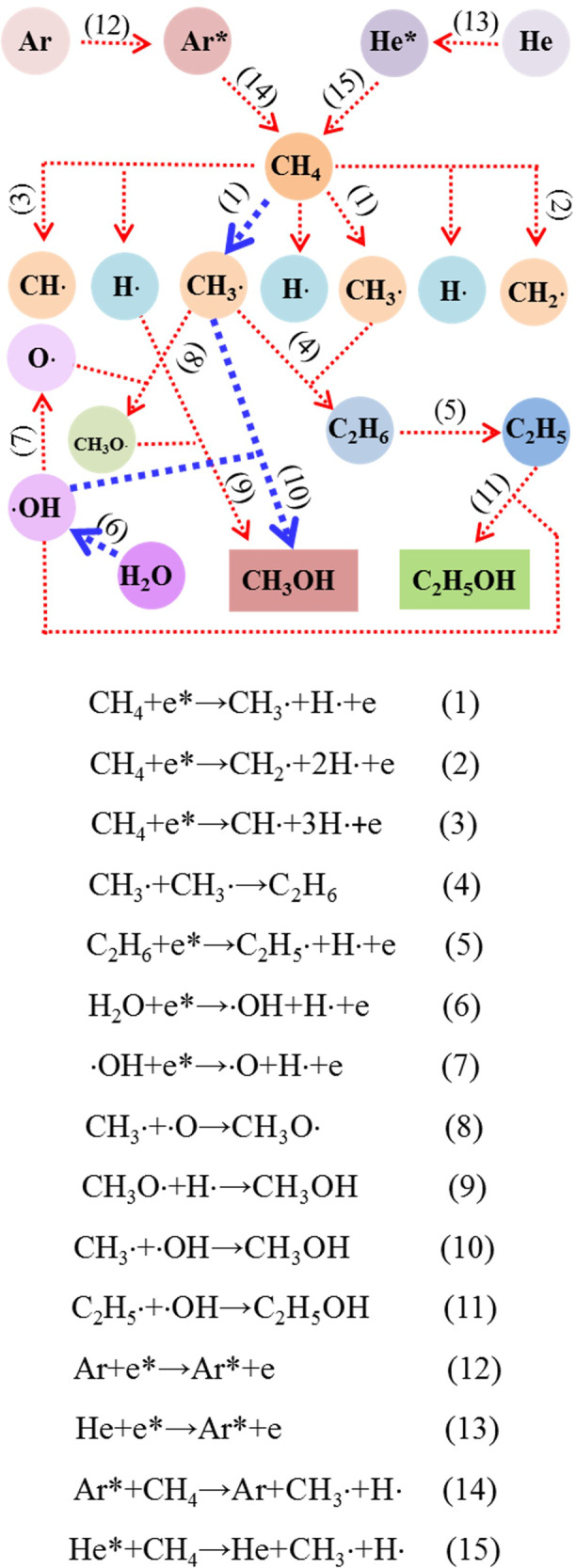
Possible reaction pathways for the reaction between CH_4_ and H_2_O with DBD. The methanol produced by the reaction mainly originates from the combination of methyl radicals (CH_3_·) and hydroxyl radicals (·OH).

**Fig. 5:**
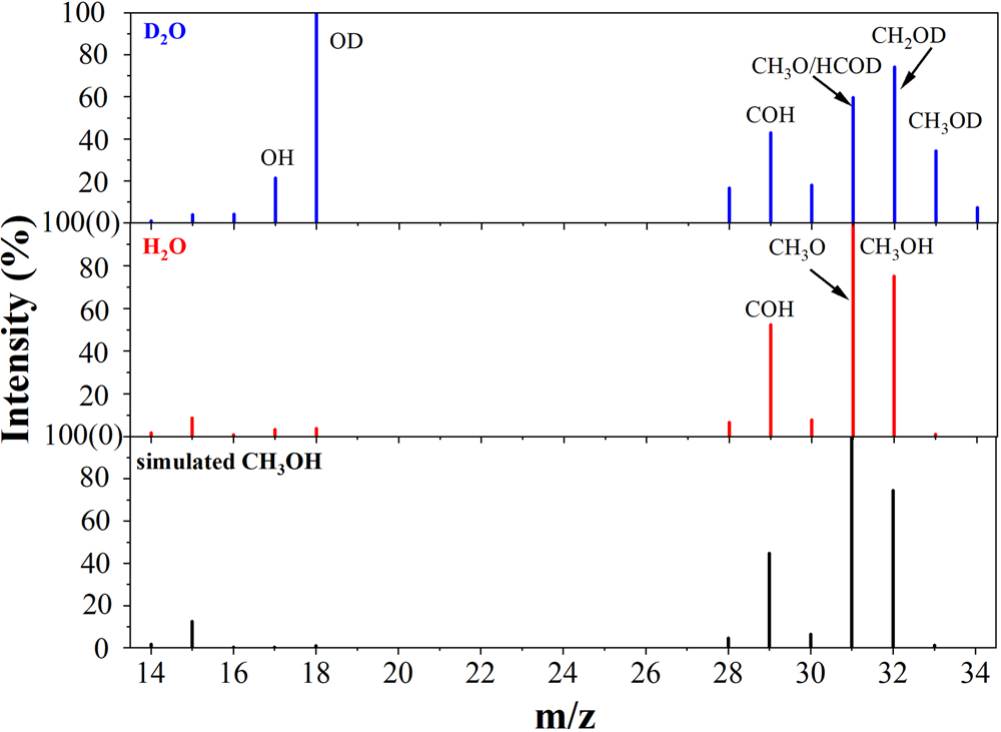
GC-MS spectra of methanol formed using H_2_O and D_2_O. The GC-MS spectra reveal that most of the produced methanol is CH_3_OD and that few molecules are CH_3_OH.

Figure 4 shows the possible reaction pathways for the formation of methanol and ethanol. Isotope tracer experiments were performed with D_2_O and analyzed by gas chromatography-mass spectrometry (GC-MS) (Fig. 5). The GC-MS spectra reveal that most of the produced methanol is CH_3_OD and that few molecules are CH_3_OH. The results reveal that the methanol produced by the reaction mainly originates from the combination of methyl radicals (CH_3_·) and hydroxyl radicals (·OH) (Eq. 10). Moreover, few CH_3_· radicals combine with ·O to form CH_3_O· and then combine with H· to form CH_3_OH (Eqs. 7-9). Experimental results show that in addition to CH_3_OH, C_2_H_5_OH is a main by-product. Therefore, increasing the CH_3_OH production rate and making efficient use of CH_3_· and ·OH are the key issues for improving methanol production rate and selectivity.

### Effect of adding inert gas on methanol production rate

**Fig. 6:**
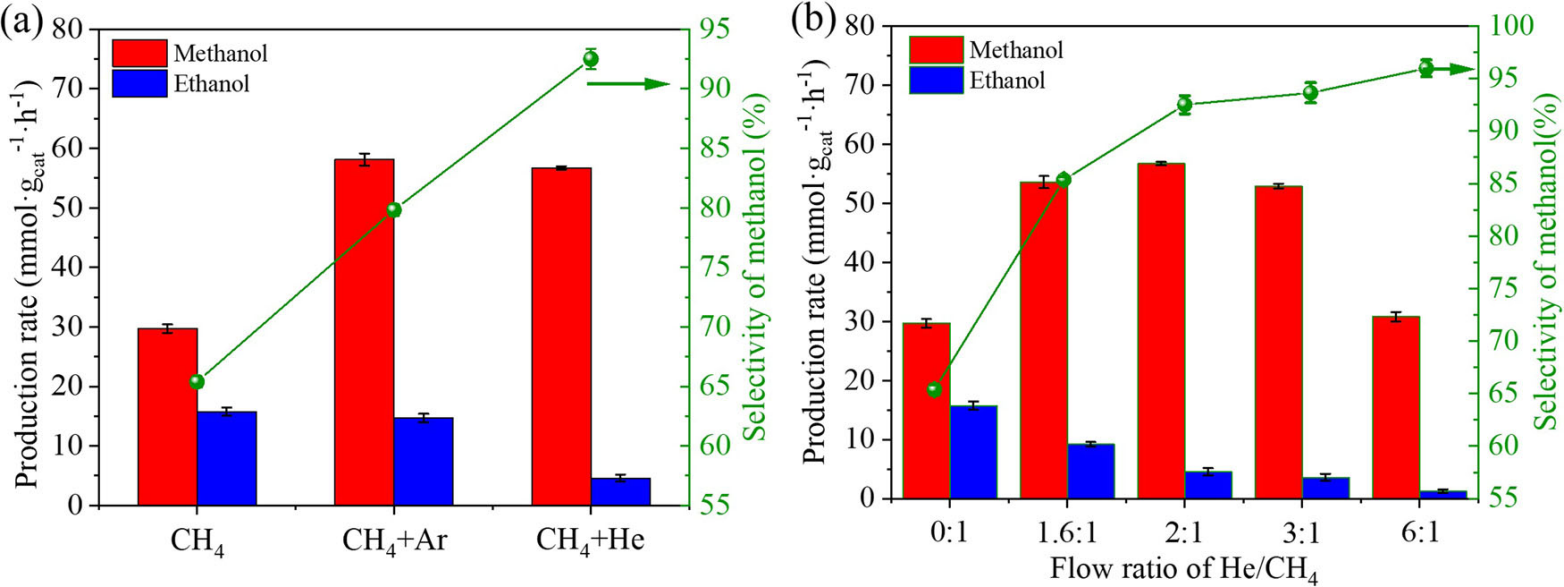
Alcohol formation rate and methanol selectivity. **a** Effect of the addition of He or Ar on the reaction at total flow rate of 15 mL min^−1^. **b** Effect of the He/CH_4_ feed ratio on the reaction at CH_4_ flow rate 5 mL min^−1^ (discharge power of 30 W, ca. 5 mg TiO_2_-700 catalyst, error bars obtained from repeated three sets of experiments on the same catalyst).

The addition of an inert gas (e.g., Ar, He) to the reaction feed to improve the reaction performance was explored from two aspects. On the one hand, in DBD plasma, CH_3_· radicals constitute the main free radicals produced from CH_4_^23^, but other free radicals are also present (e.g., CH_2_· and CH·) (Eqs. 1–3). At this time, due to the dilution of the original radical composition by Ar or He, the self-coupling of methyl radicals is reduced (Eq. 4). Hence, the produced methyl radicals are utilized for methanol synthesis to a greater extent, and the selectivity of methanol is subsequently considerably improved. On the other hand, the added Ar or He gas can produce metastable atoms with a longer average life span, thereby leading to Penning ionization. Ar or He activated by electrons transfers its energy to CH_4_ for the activation of an increased number of CH_4_ molecules^24^ (Eqs. 15, 16). The formation of CH_3_· from CH_4_ in a plasma is caused by the energetic electron-initiated dissociation of CH_4_ (bond dissociation energy: 4.52 eV). Furthermore, ·OH can be formed by the electron impact dissociation of H_2_O (bond dissociation energy: 5.19 eV). Therefore, the average electron energy in a DBD plasma is a key factor affecting the reaction. The electrons in the high-energy tail in the Maxwellian distribution are responsible for the dissociation of CH_4_ and H_2_O. Introducing He or Ar can increase the average electron energy in the reactor, which leads to higher CH_4_ and H_2_O conversions. The addition of Ar or He considerably increased the production rate of methanol. With the addition of Ar, the production rate reached 58.1 mmol g_cat_^−1^ h^−1^ (Fig. 6a). Compared to previous studies, this system exhibits an excellent methanol production rate, and most of the previous studies used O_2_ or H_2_O_2_ as the oxidant (Table 1). With the addition of He, the production rate reaches 56.7 mmol g_cat_^−1^ h^−1^. Although the production rate increase with He is slightly lower than that with Ar, the selectivity of methanol clearly increases from 65 to 93% in the liquid products under CH_4_/He plasma. After the addition of Ar or He, the plasma discharge current waveform was monitored (Supplementary Fig. 3). The addition of Ar increases the electron density^25^, which in turn increases the collision probability between electrons and CH_4_ in the reaction. As a result, an increased number of CH_4_ molecules are activated and transformed (Supplementary Fig. 4). The addition of He leads to an increase in the average electron energy in the plasma region but reduces the electron density of the system^26^. Hence, the CH_4_ conversion increase is less than that with the addition of Ar.

**Table 1:** Comparison of methanol production rates obtained in this study and in previous studies.

Catalyst	Conditions	Oxidant	Methanol production rate	Ref.
TiO_2_	35 °C, 1 bar 30 W, Plasma	H_2_O	56.7 mmol g_cat_^−1^ h^−1^	This work
AuPd@ZSM-5-C_16_	70 °C, 30 bar	O_2_	91.6 mmol g_AuPd_^**−**1^ h^**−**1^	^7^
Cu-MOR, 4.7 wt% Cu	multi-step reaction 450 °C, 1 bar O_2_ 200 °C, 36 bar CH_4_	O_2_	0.3 mmol g_cat_^**−**1^ h^**−**1^	^27^
0.5 wt% Rh-ZSM-5	150 °C, 4 bar O_2_ 5 bar CO, 20 bar CH_4_	O_2_, CO	1.22 mmol g_cat_^**−**1^ h^**−**1^	^28^
0.5 wt% Fe-S-1&Cu/S-1	70 °C, 3 bar	H_2_O_2_	6.2 mmol g_cat_^**−**1^ h^**−**1^	^29^
Cr/ TiO_2_	50 °C, 30 bar	H_2_O_2_	0.34 mmol g_cat_^**−**1^ h^**−**1^	^30^
0.3 wt% Rh-ZrO_2_	70 °C, 30 bar	H_2_O_2_	25.5 mmol g_Rh_^**−**1^ h^**−**1^	^31^
0.33_metal_ wt% FeO_*x*_/TiO_2_	25 °C, 1 bar 300 W, Xenon lamp	H_2_O_2_	0.65 mmol g_cat_^**−**1^ h^**−**1^	^32^

### Effect of the flow ratio of He/CH_4_ on the methanol production rate

The addition of He improves methanol production rate as well as methanol selectivity. Next, the effects of the addition of different amounts of He on the methanol production rate and selectivity were investigated (Fig. 6b). The results reveal that the selectivity of methanol increases with the increase in He addition. At a He/CH_4_ ratio of 6:1, the selectivity of methanol reaches 96%. Simultaneously, with the increase in the He/CH_4_ feed ratio, the methanol production rate initially increases and reaches a peak at a He/CH_4_ feed ratio of 2:1. A further increase in the He/CH_4_ ratio leads to a decrease in methanol production rate due to a possible decrease in the gas–liquid contact time by the excess airspeed.

In conclusion, a new route for the direct conversion of methane to methanol in one step at room temperature under atmospheric pressure was designed and demonstrated with simple operation. The entire reaction was further tailored by considering two aspects: CH_3_· production and ·OH production. A methanol production rate of 56.7 mmol g_cat_^−1^ h^−1^ and a selectivity of 93% in the liquid products were obtained, and CO_2_ formation was not observed, successfully inhibiting the over-oxidation of methane. This designed reaction system may also be applied for the selective oxidation of other stable chemicals such as benzene to phenol.

## Methods

### Catalyst preparation

Purchased TiO_2_ powders were ground in an agate mortar, then placed in a crucible and transferred to a muffle furnace. The TiO_2_ samples were heat treated in an air atmosphere at 500 °C, 600 °C, 700 °C, or 800 °C for 4 h to obtain a series of catalysts, which were recorded as TiO_2_-t, where t is the calcination temperature.

### Catalyst characterization

Powder X-ray diffraction (XRD) patterns were measured using a Rigaku Smart Lab diffractometer, with a nickel-filtered Cu Kα X-ray source, at a scanning rate of 0.02° over a 2θ range of 5° to 80°. Transmission electron microscopy (TEM) tests were performed using a Tecnai G2 20 S-twin instrument (FEI Co.) with an accelerating voltage of 200 kV. TEM samples were ultrasonicated in ethanol, dropped onto carbon-coated copper mesh, and dried under ambient conditions. High-resolution transmission electron microscopy (HRTEM) measurements were obtained to determine the lattice spacing of the TiO_2_ for a comprehensive understanding of the crystal structure. X-ray photoelectron spectroscopy (XPS, Thermo Scientific K-Alpha), with an Al Kα excitation source (*hν* = 1486.6 eV), was used to determine core level binding energies of surface species. Sample charging was corrected by referencing all measurements to the C (1s) peak at 284.8 eV. Raman spectra were recorded with a Horiba Scientific LabRAM HR Evolution spectrograph using 532 and 325 nm laser lines. UV–Vis absorption spectra were obtained in the range of 200–800 nm using a UV-2600 spectrophotometer. The methanol product in the isotope-labeled reaction was analyzed by a gas chromatograph-mass spectrometer (GCMS) using an Agilent GCMS 7890B-5977B equipped with an HP-5ms chromatographic column.

### Experimental

Supplementary Fig. 5 shows a process flow diagram of the reaction system. 5 mg catalyst was ultrasonically dispersed in 3 mL H_2_O and placed in a dielectric barrier discharge (DBD) reactor. The details of the plasma reaction system are shown in Supplementary Fig. 6. The methane feed into the reactor was controlled by a mass flow meter at a constant rate of 5 mL min^−1^. The plasma was generated using a high-voltage power supply, and the reaction was allowed to proceed for 20 min. The plasma power (30 W) is the input power and is calculated by multiplying the input voltage (50 V) and the input current (0.6 A). In addition, a Lissajous figure was measured with 30 W input power using an oscilloscope. Supplementary Fig. 7 shows the Lissajous figure at an input power of 30 W. According to Supplementary Fig. 7, the actual output power corresponding to the input power of 30 W can be calculated to be 22.4 W. The applied voltage peak to peak (Vpk-pk) was approximately 27 kV (Supplementary Fig. 8). The reaction solution was filtered and then analyzed by gas chromatography to determine the product composition. Methanol and ethanol were analyzed by a gas chromatograph (GC) equipped with an FID detector and a Porapak Q column. The standard calibration curves of the peak areas and methanol and ethanol concentrations are shown in Supplementary Figs. 9 and 10. Methane was analyzed using a GC equipped with an FID detector and a 30 m PLOT-Q capillary column. CO_2_ was analyzed using a TCD detector and a TDX-01 molecular sieve column.

### Calculation method of discharge power

We calculated the average power by finding the area under the curve for the *V*–*Q* Lissajous plot and multiplying it by the frequency.

$$P=\oint v\left(t\right)\times q\left(t\right)\times {{{{{{\rm{d}}}}}}t}=\frac{f}{2\pi }\times S$$

where *P* is the average power in *W*, *v*(*t*) is the voltage measured by the oscilloscope, *q*(*t*) is the charge measured by the oscilloscope in *C*, *f* is the frequency in kHz, and *S* is the area under the curve for one cycle.

## Supplementary information

Supplementary Information

## Data Availability

The authors declare that the/all other data supporting the findings of this study are available within the paper and its supplementary information files.
