# Supplementary material for: One-step direct conversion of methane to methanol with water in non-thermal plasma
Source: Commun Chem. 2022 Oct 10;5:124. doi: 10.1038/s42004-022-00735-y (PMC9814404; doi:10.1038/s42004-022-00735-y)
Supplement: Supplementary file 1 — Supplementary Information [file 42004_2022_735_MOESM1_ESM.pdf]

Supplementary Information

One-step direct conversion of methane to methanol with water in non-thermal plasma

Wenfei Bi[1], Yu Tang[1], Xuemei Li[1], Chengyi Dai[1]*, Chunshan Song[2], Xinwen Guo[3], Xiaoxun Ma[1]

The conversion of methane was calculated using the following equation:

$$Conversion\,(\%) = \frac{mol_{\text{initial methane}} - mol_{\text{methane after reaction}}}{mol_{\text{initial methane}}} \times 100\%$$

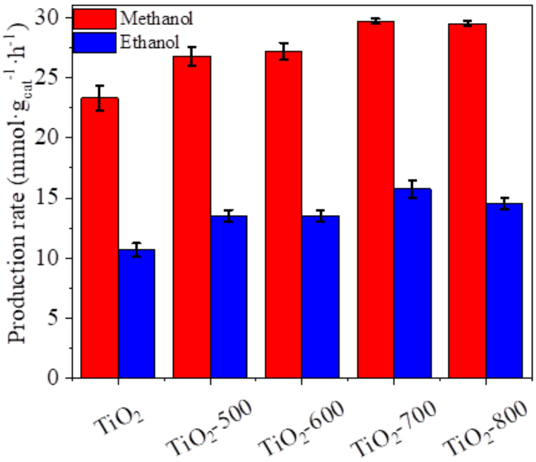

Supplementary Figure 1. Effects of different catalysts on the reaction (error bars obtained from repeated three sets of experiments on the same catalyst).

Supplementary Table 1. Selectivity of gas phase and liquid phase products.

Entry	Gas phase product selectivity (%)						Liquid phase product selectivity (%)					
	C_2H_4	C_2H_2	C_2H_6	C_3H_6	C_3H_8	CO	HCHO	HCOOH	CH_3CHO	CH_3COOH	CH_3OH	CH_3CH_2OH
1	1.6	1.2	64.0	0.2	16.9	4.8	trace	trace	trace	trace	4.5	6.7
2	2.2	1.7	56.0	0.5	22.9	1.9	trace	trace	trace	trace	7.7	7.1
3	1.3	1.3	49.9	0.4	17.6	14.1	trace	trace	trace	trace	10.2	5.1
4	0.9	0.7	54.7	0.2	21.0	7.6	trace	trace	trace	trace	12.7	2.0

Entry 1: No catalyst, No gas added

Entry 2: TiO_2-700, No gas added

Entry 3: TiO_2-700, Ar

Entry 4: TiO_2-700, He

After the reaction, the gas phase product and the liquid phase product are easily separated, and the liquid phase product is mainly methanol. Although some by-products are inevitably generated in the gas phase, they have a higher added value relative to methane. Further, separation can be achieved by a chemical reaction, for example using gas phase products directly for an alkylation reaction.

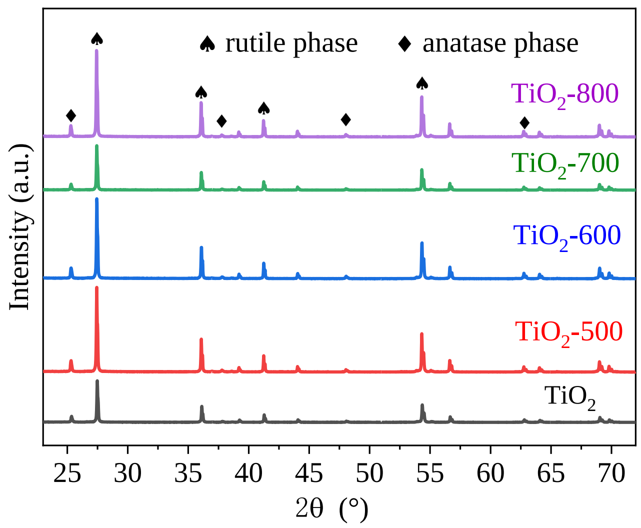

Supplementary Figure 2. XRD of TiO_2 after calcination at different temperatures from 500 to 800 °C.

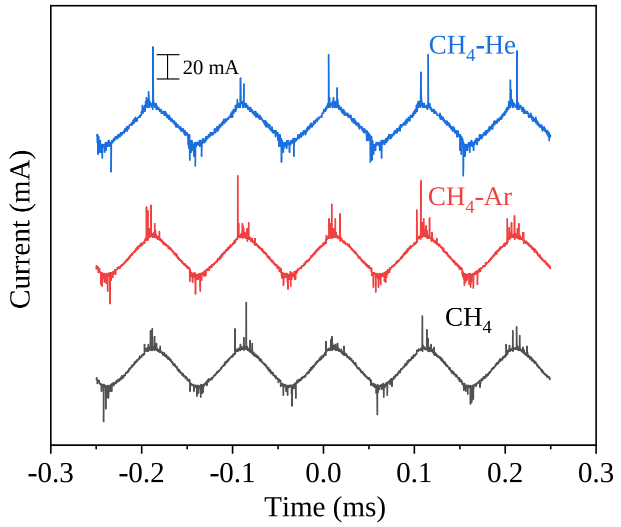

Supplementary Figure 3. Discharge current waveform after adding Ar or He.

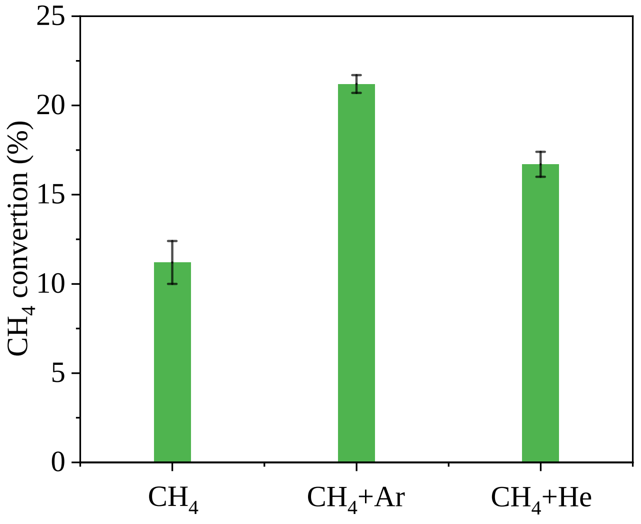

Supplementary Figure 4. The effect of adding Ar or He on methane conversion (error bars obtained from repeated three sets of experiments on the same catalyst).

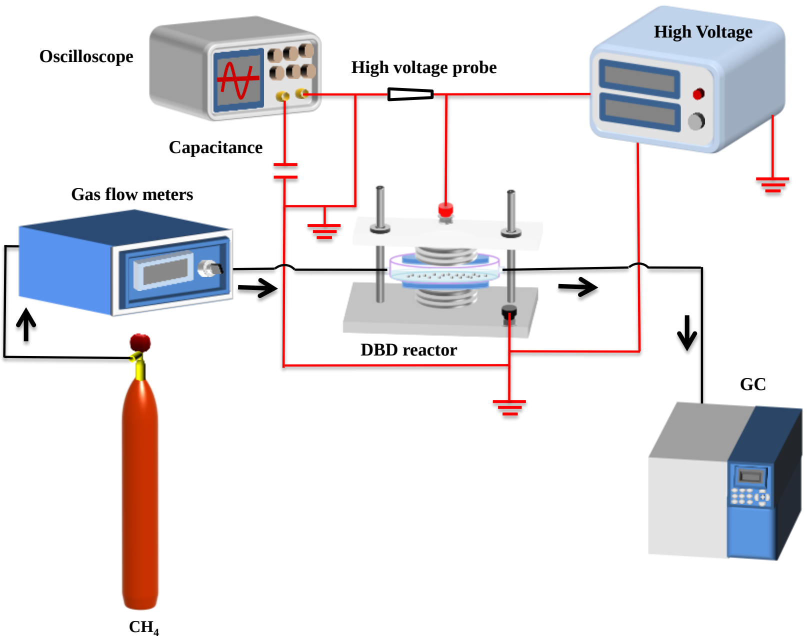

Supplementary Figure 5. Schematic diagram of experimental setup.

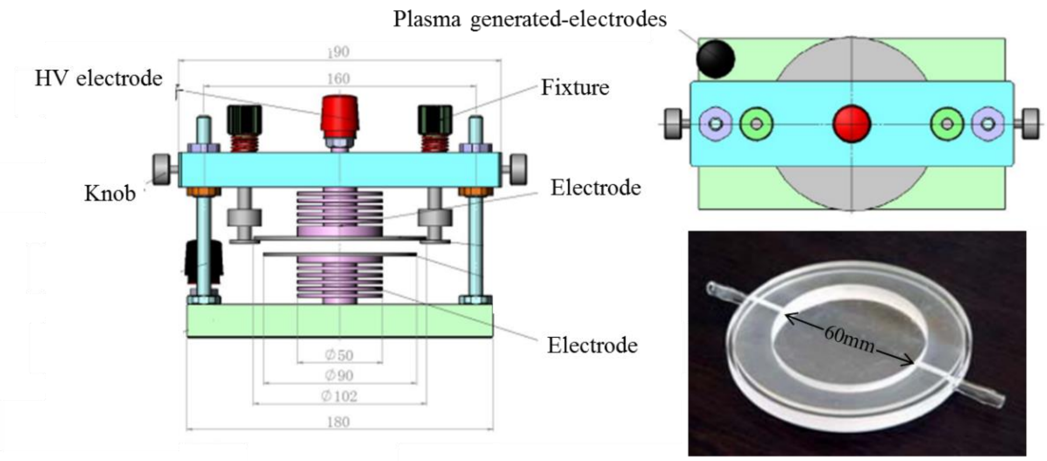

Supplementary Figure 6. Dimensions of the plasma reaction system.

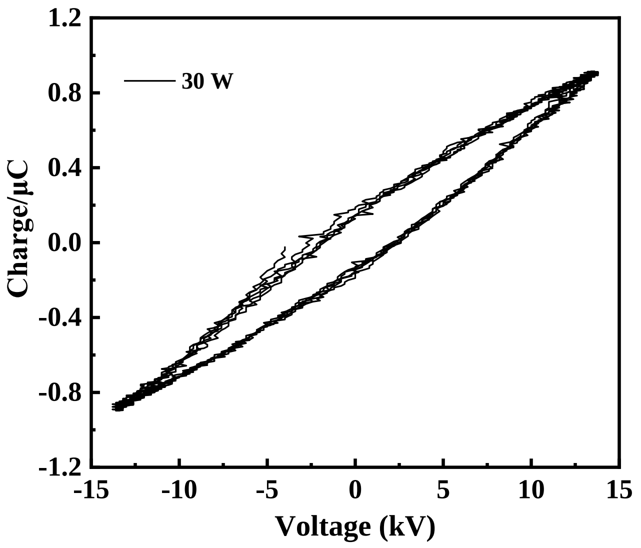

Supplementary Figure 7. Lissajous figures at 30W of input powers.

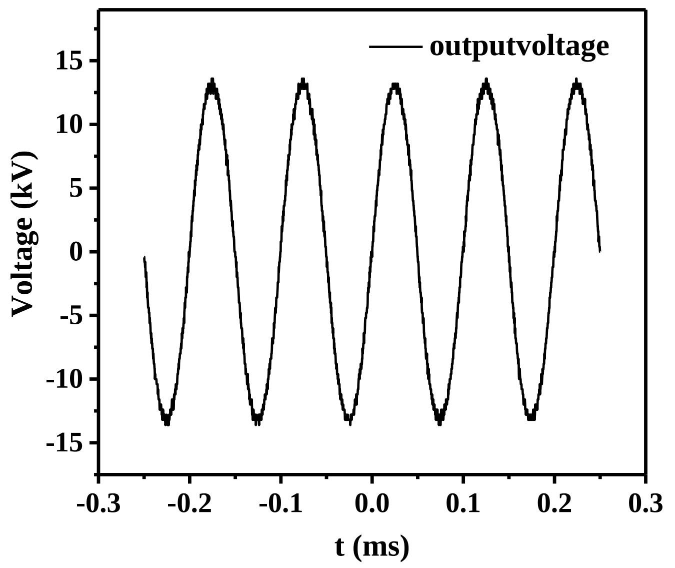

Supplementary Figure 8. The output voltage of the plasma discharge device when the input power is 30W.

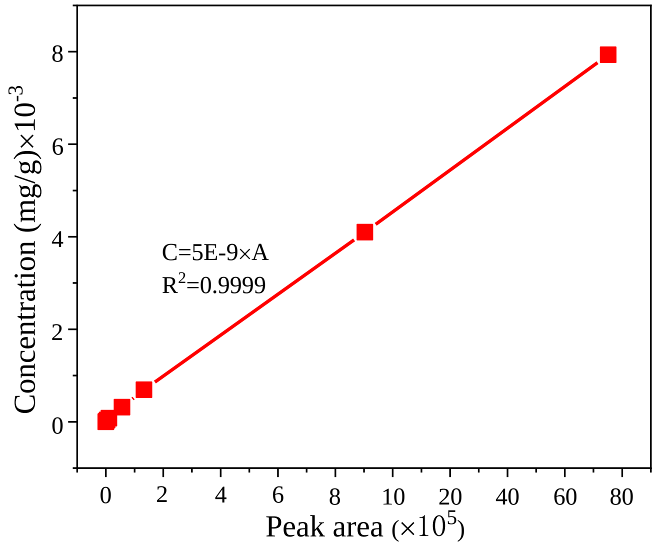

Supplementary Figure 9. The standard calibration curve of peak area and methanol concentration.

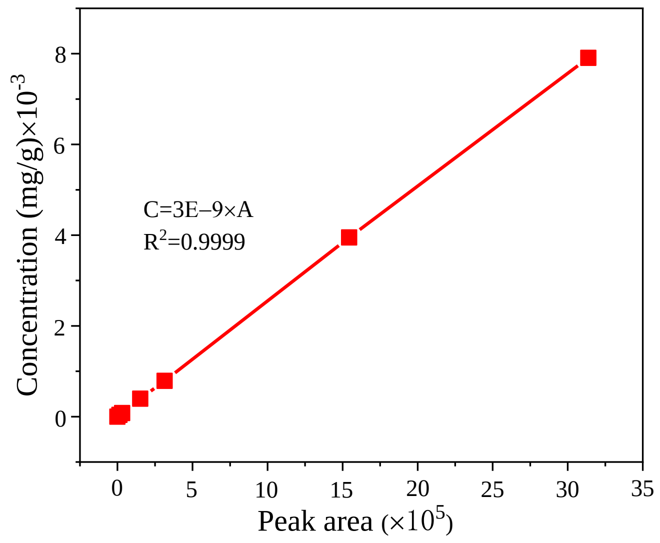

Supplementary Figure 10. The standard calibration curve of peak area and ethanol concentration.
